# National Health Insurance Notes

**Published:** 1923-03

**Authors:** 


### NATIONAL HEALTH INSURANCE NOTES.

### THE PREVENTION OF SICKNESS.

SIR WALTER KINNEAR, in a recent address to Approved Societies' Secretaries, did well to draw attention to the fact that the National Health Insurance scheme included the prevention as well as the cure of sickness. The Regional Medical Officers, he said, had examined the records of 36,000 cases of illness. As a result of their analysis they found that the majority of insured persons suffered from maladies of which a great proportion were preventable. He urged secretaries to get away from the idea that they were only there to pay cash benefits. He advised them not to forget Section 63, which had not really been put in operation so far; and to consider the machinery of that section, not simply from the point of view of securing a refund of benefit paid out, but with a view to removing the disabilities and disadvantages under which insured persons laboured. This section of the Act is the one designed to enable Societies and Committees to discover those black spots of the country where sickness breeds, and to take steps to wipe them out. It is worth the while of all concerned to devote their energies to making use of such powers as they possess for the prevention of sickness.

### THE DOCTOR'S ASSISTANT.

In so much of the criticism of the provision which enables an insurance doctor, with the consent of the Insurance Committee, to engage the services of an assistant in his practice among insured persons, there is to be observed that failure to distinguish between the principle involved and its abuse which is to be noticed in other criticisms of the panel or any other system. The patients of a doctor are entitled at any time to require his personal services, and he must give them unless prevented by some reasonable cause. If a doctor who has an assistant leaves the care of his insurance patients as a matter of course to his assistant, and is as a rule himself inaccessible to those patients, that is clearly an abuse of the system which has got to be dealt with. It can be dealt with under the Regulations. But to demand, therefore, that doctors should not be allowed to employ assistants is to display a singular lack of perception. Is the young practitioner likely to make more or fewer mistakes if working in close daily consultation with the experienced doctor? Is this training-ground among one-third of the whole population to be barred to him? If so, what of the future of the medical services of the country?

### A NEGLIGIBLE RISK.

It is, perhaps, only human that many doctors should be uneasy about the risk which they appear to run of giving free treatment to persons who can produce no evidence of title, and are not, in fact, entitled to such treatment. The figures quoted at the Guildhall Conference by Sir William Glyn-Jones ought to reassure them. The doctor is, of course. allowed to receive a fee by way of returnable deposit in cases of doubt. It appears that in the county of Middlesex, in the course of a year, 757 such deposit payments were made, and in 37 cases only was the applicant found not to be entitled to receive medical treatment as an insured person. This must be an entirely negligible fraction of the total insured population of the county. Again, in Liverpool, out of 493 cases in which deposit receipts were given, the title to benefit was established in all but four cases.

### IS HE AN INSURED PERSON?

This is a question which some doctors will have you believe they feel great delicacy in asking some patients. Why this should be so we are at a loss to imagine. Assuming for the moment that it is a delicate question, surely it is part of the equipment of every doctor to be able to ask delicate questions nicely. But, as a fact, there should be no difficulty about it. Every manual worker who is not his own master is insured, and every insured person is, without other qualification, entitled to medical benefit. The central fund out of which the doctors are paid is based on an actuarial estimate of the insured population as a whole, and its amount is not in any way dependent upon the declarations which the insured persons make in doctors' surgeries. The case which presents any real doubt is that of the clerical or other non-manual worker whose remuneration may be more or less than £250 a year. But no one really expects the doctor to ask such a person what his income is, although this was seriously suggested at the Guildhall Conference. Of course, if the applicant says "I am insured, but I want to be treated as a private patient," the doctor should be for his part capable of making it clear that he is not in partnership with himself, that he cannot in this way distinguish between private and insured patients, and that he does not want to be paid twice over for the same service.

### THE COST OF CHECKING EXCESSIVE PRESCRIBING.

It is permissible to ask, as at present is being done, whether the cost of getting out schedules of prescriptions for the use of Panel Committees in connection with their duties in checking extravagant prescribing is justified by the results. But it is not a proper answer to add up the amounts of penalties imposed on certain doctors and point out that it falls short of the cost of getting out the particulars. The main object of the check is secured if doctors, knowing that there will be a scrutiny, exercise reasonable care in prescribing. The Panel Committee had a good excuse for slackness in carrying out their duties so long as they were supposed to wade through the prescriptions themselves. They have no longer that excuse. But it may well be that the preliminary work of examining the prescriptions and clearing up most of the points which arise for enquiry would be more effectively done, and with less irritation to the individual doctors, if taken in hand by the Regional Medical Officer, leaving to the Panel Committee the judicial functions appertaining to the cases requiring a formal investigation. We shall be interested to see the nature of the proposals foreshadowed by the Ministry some time ago.

